# A Comparison of Marine and Non-Marine Magnesium Sources for Bioavailability and Modulation of TRPM6/TRPM7 Gene Expression in a Caco-2 Epithelial Cell Model

**DOI:** 10.3390/nu18020324

**Published:** 2026-01-20

**Authors:** Olusoji A. Demehin, Michelle Ryan, Tommy Higgins, Breno Moura Motta, Tim Jähnichen, Shane O’Connell

**Affiliations:** 1 Marigot Ltd., Marigot Research Centre, Sycamore Court, V92 N6C8 Tralee, Ireland; michelle.ryan@marigot.ie (M.R.); tommyhiggs@gmail.com (T.H.); shane.oconnell@marigot.ie (S.O.); 2 Centre for BioNano Interactions, School of Chemistry, University College Dublin, D04 N2ES Dublin, Ireland; breno.motta@cbni.ucd.ie; 3 School of Chemistry, University College Dublin, D04 N2ES Dublin, Ireland; tim.jahnichen@ucd.ie; 4 Centre for Applied Bioscience Research, Munster Technological University-Tralee, South Campus, Clash, V92 CX88 Tralee, Ireland

**Keywords:** Aquamin Mg Soluble, magnesium bioavailability, Caco-2, TRPM6, TRPM7, gastrointestinal digestion

## Abstract

Background/Objectives: Magnesium (Mg^2+^) supplements can contain different types of Mg^2+^ salts, which influence their bioavailability. A highly bioavailable and bioaccessible Mg^2+^ source is essential to meet requirements for many physiological processes that are fundamental to human health. The objective of this study was to compare the bioavailability of Mg^2+^ from different sources, with different composition and chemical structure, namely, Aquamin Mg Soluble (seawater), magnesium oxide, commercial magnesium bisglycinate 1, and analytical grade magnesium bisglycinate 2. In addition, the influence of the different Mg^2+^ sources on transported Mg^2+^ and expression of *TRPM6* and *TRPM7* genes in Caco-2 cell monolayers was also evaluated to estimate bioavailability. *TRPM6* and *TRPM7* are members of the transient receptor potential melastatin subfamily characterized as Mg^2+^ permeable channels. Method: The study involved analyzing bioavailability of the Mg^2+^ sources predigested with and without food using the Infogest model prior to application to a Caco-2 cell monolayer in transwells for assessing transport. Mg^2+^ concentration on the basolateral side was analyzed by ICP-MS, and expression of *TRPM6* and *TRPM7* genes in the monolayer was analyzed using real-time qPCR. Results: Aquamin Mg Soluble showed significantly higher bioavailability compared to magnesium bisglycinate 2 (*p* = 0.016) when digested with food prior to application to the Caco-2 monolayer. In the digestion without food prior to the Caco-2 monolayer, there was no significant difference between Mg^2+^ bioavailability among the tested supplements. The *TRPM6* gene was significantly downregulated in Caco-2 monolayers exposed to Aquamin Mg Soluble compared to untreated Caco-2 cells (*p* < 0.001). Conclusions: The INFOGEST digestion model showed that Aquamin Mg Soluble provides a highly bioavailable form of Mg^2+^, while the Caco-2 monolayer model also demonstrated its increased bioavailability by the modulation of TRPM6 gene expression.

## 1. Introduction

In the body, Mg^2+^ functions as a co-factor for over three hundred enzymes responsible for regulating multiple physiological processes, including energy production, protein synthesis, neuromodulation, regulation of blood glucose, and blood pressure [1]. Certain foods, such as seeds, fish, whole grains, green leafy vegetables, and nuts, are examples of natural sources of good quantities of dietary Mg^2+^. Nevertheless, studies have shown that Mg^2+^ intake is inadequate around the world [2]. The prevalence of Mg^2+^ deficiency as reported by studies up to 2018 was approximately 15–20% of the population in developed countries [2,3,4]. Hypomagnesemia, a condition characterized by serum Mg^2+^ levels below 1.46 mg/dL (0.6 mmol/L), has been associated with the occurrence of type-2 diabetes mellitus, disorientation, confusion, neuromuscular irritability, tremors, anxiety, depression, osteoporosis, and heart failure [2,5,6,7]. While multiple factors may contribute to Mg^2+^ deficiency, low bioavailability of dietary Mg^2+^ is a major cause. Therefore, dietary supplements are an important tool to help prevent Mg^2+^ deficiency. Manufacturers use diverse forms of Mg^2+^ for supplement manufacture, with Mg^2+^ sources including organic and inorganic salts, e.g., oxide, citrate, chloride, gluconate, or lactate, and glycinate, with significant variation in the absorption potential of Mg^2+^ depending on the source [8,9,10,11,12,13].

Magnesium bioavailability studies in humans and animals are time-consuming to perform and expensive; thus, physiologically relevant in vitro models are increasingly used to assess potential bioavailability. The use of Caco-2 cell monolayers in transwells for bioavailability studies has been frequently used by researchers in recent years [14]. However, inappropriate design of in vitro bioavailability studies could limit the ability of the model to serve as an accurate predictor of Mg^2+^ bioavailability in vivo in humans. For example, an in vitro study by Kyselovič et al., 2021 [9] involving the use of the Caco-2 cell model showed that magnesium citrate had significantly lower Mg^2+^ transport compared with magnesium oxide over 2 h. In contrast, clinical studies have demonstrated higher Mg^2+^ bioavailability for magnesium citrate compared to magnesium oxide in human subjects [10,11]. It is important to note that the magnesium supplements tested in Kyselovič et al., 2021 [9] were not subjected to simulated gastrointestinal digestion prior to exposure to Caco-2 cell monolayers. This approach does not replicate the physiological conditions of the human gastrointestinal tract, where Mg^2+^ salts undergo physicochemical transformation during digestion. In contrast, in vitro bioavailability studies for Mg^2+^ [8] and Iron [15] that incorporated a digestion step prior to Caco-2 cell exposure have reported results consistent with clinical findings. Therefore, the harmonized INFOGEST in vitro digestion protocol [16], which accounts for digestive enzymes, pH, time, and digestive fluids, was incorporated into the present study. The integration of digestion steps improves physiological relevance and allows mechanistic investigation of epithelial nutrient transport.

Physiological regulation of Mg^2+^ relies on highly bioaccessible Mg^2+^ from supplements or diet, intestinal absorption, renal excretion, and exchange with bone. Intestinal absorption of Mg^2+^ involves two different transport systems acting in parallel: An active transcellular and a passive paracellular pathway [17]. Transcellular uptake involves apical Mg^2+^ entry through specific channels^,^ followed by basolateral extrusion via mechanisms that remain incompletely defined [18]. The genes involved in this process include *TRPM6* and *TRPM7*, members of the transient receptor potential melastatin (TRPM) family, which regulate Mg^2+^ and other cation permeation [19]. *TRPM7* functions as a Mg^2+^ sensor and transducer, and is essential for cell proliferation, differentiation, growth, and migration [20,21]. Investigation into the relationship between cellular Mg^2+^ and *TRPM7* by Schmitz et al., 2003 [22] revealed that TRPM7-deficient cells become Mg^2+^ deficient and that the viability and proliferation of the cells were rescued through extracellular Mg^2+^ supplementation. *TRPM6* has been described to be expressed abundantly in epithelial cells, especially in colon and kidney cells, where it forms a channel on the apical membrane [23] to allow Mg^2+^ influx and functions as a channel for Mg^2+^ entry [21]. Animal studies have shown that dietary Mg^2+^ restriction leads to increased *TRPM6* expression in the colon, suggesting a compensatory response to low Mg^2+^ availability [24,25,26].

Although Felice et al. (2018) [8] previously compared a marine-derived magnesium source (Aquamin Mg Soluble) with commercially available magnesium oxide and chloride, comparative data with organic magnesium sources such as magnesium glycinate remain limited, particularly under simulated digestive conditions. Moreover, previous work did not evaluate Mg^2+^ bioavailability and epithelial transport in the presence of food, which may influence Mg^2+^ solubility through interactions with other minerals and biomolecules in the digestive tract. Aquamin Mg Soluble is a natural seawater-derived Mg^2+^ source that contains 72 trace minerals [27], and the complex composition and physicochemical properties [28] may influence marine-derived Mg^2+^ behavior following digestion. Therefore, the objective of this study was to compare transport of Mg^2+^ from Aquamin Mg Soluble, magnesium oxide, and two magnesium glycinates after INFOGEST digestion with and without food using Caco-2 monolayers in transwells. In addition, the study examined changes in *TRPM6* and *TRPM7* gene expression as indicators of cellular responses related to Mg^2+^ transport under in vitro conditions.

## 2. Materials and Methods

### 2.1. Composition and XRD Analysis of Inorganic Magnesium Sources

The total inorganic content of the Mg^2+^ sources was determined using a gravimetric method. Briefly, 1 g of dry sample was ashed in a muffle furnace at 500 °C for 5 h. The percentage inorganic matter was calculated as the ratio of ash to initial weight of the dry sample. Mg^2+^ content was measured by digesting triplicates of 200 mg of Mg^2+^ sources in 10 mL 65%*v*/*v* nitric acid. Digestion was performed in a microwave digester at 190 °C for 20 min. Mg^2+^ concentration was measured using an Agilent ICP-MS 7800 instrument (Agilent Technologies Ireland Ltd., Cork, Ireland) tuned using Agilent Tuning Solution for ICP-MS (1 ppb), part No. 5185–5959, and calibrated with Agilent Environmental Calibration Standard, part No. 5183–4688. Mg^2+^ calibration curves had r-squared values > 0.98. XRD analysis was performed by mounting the Mg^2+^ samples on a low-background sample holder. Powder X-ray diffraction patterns were obtained on a Rigaku Miniflex 600 X-ray diffractometer, Rigaku, Tokyo, Japan, using CuKa radiation (k = 1.5418 Å), from 5 to 60°, with steps of 0.02°. Measurements were taken at the X-ray & SQUID lab, School of Chemistry, UCD.

### 2.2. Surface Area Analysis

The sample powders were degassed and heated to 150 °C for 24 h using the micromeritics VacPrep 061 sample degas system. Surface area was determined by nitrogen adsorption measurements carried out using the micromeritics 3Flex at 77 K. Obtained data was analyzed using Brunauer-Emmelt-Teller (BET) theory on the 3Flex software v6.03.

### 2.3. In Vitro Bioavailability Method

#### 2.3.1. In Vitro Digestion of Mg^2+^ Sources

The Mg^2+^ sources were subjected to the INFOGEST digestion simulation prior to application to Caco-2 cell monolayers on transwells. Three independent digestions were performed for each source under two approaches: digestion of the Mg^2+^ source with food and without food material. The quantity of each Mg^2+^ source corresponding to 60 mg Mg^2+^ was digested according to the harmonized INFOGEST in vitro digestion model published by Brodkorb et al., 2019 [16]. A control sample was prepared, following the same digestion protocol but excluding the Mg^2+^. At the end of the digestion, samples were taken for bioavailability study using Caco-2 cell monolayers on transwells.

#### 2.3.2. Caco-2 Cell Monolayer Model

A Caco-2 cell monolayer in transwells was used for the in vitro evaluation of Mg^2+^ bioavailability after subjecting the tested Mg^2+^ sources to a simulated digestion process. Caco-2 cells were obtained from ECACC—catalogue number 09042001 and used in experiments at passage P51 to P79. Caco-2 cells were grown in DMEM (GIBCO) with 10% (*v*/*v*) heat-inactivated fetal calf serum (GIBCO), 4 mM L-glutamine (GIBCO), 2 mM sodium pyruvate (GIBCO), 1% non-essential amino acids (GIBCO). To study Mg^2+^ transport across an epithelial barrier, 10^5^ Caco-2 cells were seeded on a polyethylene terephthalate (PET) transwell (Thermo Fisher Scientific Ireland, Cork, Ireland) insert in 6-well plates. To initiate differentiation, the plates were incubated at 37 °C in an incubator with 5% CO_2_ and 95% air atmosphere at constant humidity, and the medium was changed every 2 days for 21 days. Characteristics of differentiated Caco-2 cells usually include formation of microvilli structure, brush border on the surface of cells, tight junction formation between cells, secretion of hydrolases, and synthesis of carrier transport systems for sugars, amino acids, and drugs.

#### 2.3.3. Evaluation of Barrier Integrity, Active Transport Functionality, and Mg^2+^ Bioavailability

Caco-2 barrier integrity was assessed by the addition of 100 µg/mL Fluorescein Isothiocyanate-Dextran (3KDa, D3305, Thermo Fisher Scientific) onto the apical side of the transwell containing Caco-2 cell monolayer and media. Wells with media only were also included as controls. Also, 100 µg/mL caffeine was added onto the apical side of all the wells to measure the extent of active transportation within the Caco-2 monolayer. The plate was incubated at 37 °C with 5% CO_2_ for 2 h. After the experiment, the concentration of FITC dextran on the apical and basolateral sides of the well was analyzed using a colorimetric method. A FITC-dextran standard curve was prepared in a range of 0–250 µg/mL in sterile DPBS (Gibco 141900094, Thermo Fisher Scientific Ireland, Cork, Ireland). Fluorescence reading was performed at excitation wavelength 494 nm and emission wavelength 521 nm with a Varioskan LUX multimode microplate reader (Thermo Fisher). The amount of FITC-dextran transported across the Caco-2 monolayer for each well was determined from the FITC-dextran standard curve. Caffeine concentration on apical and basolateral sides of transwells was measured to determine the active transport functionality of the Caco-2 monolayers using an ultra-high-performance liquid chromatography system coupled to a photodiode array detector (UHPLC-PDA; Shimadzu Corporation, Kyoto, Japan) according to an optimized method described by Aljofan et al., 2019 [29]. Caffeine separation was done on a C-18 RP-Thermo, 5 µm, 150 mm × 4.6 mm column coupled to a Shimadzu instrument equipped with a system controller (model SCL-40), Autosampler (model SIL-40CX3), degasser (model DGU-403), and photodiode array detector (model SPD-M40). The mobile phase was constituted with water, methanol, and acetonitrile in a ratio of 84/1/15 (%*v*/*v*/*v*), filtered through a 0.6 µm filter paper, and degassed for 20 min. Caffeine was detected on a PDA detector at 254 nm and a 1 mL/min flow rate. Caffeine concentration was calculated using a calibration curve of pure caffeine (W222402, Sigma-Aldrich, St. Louis, MO, USA) prepared in a range of 0.1 µg/mL to 1000 µg/mL with a 0.9989 correlation coefficient.

Mg^2+^ bioavailability was measured in two approaches: (1) testing bioavailability of Mg^2+^ from sources digested without food; (2) bioavailability of Mg^2+^ digested with food material. Mg^2+^ concentration was measured on an inductively coupled mass spectroscopy system (ICP-MS) as outlined in 2.1. Prior to analysis, aliquots taken from the wells were digested in a microwave digester (CEM corporation, Matthews, NC, USA) with 50%*v*/*v* water and 50%*v*/*v* nitric acid (65%*v*/*v* Nitric acid, Trace Metal grade). The digested samples were filtered and diluted appropriately with a diluent constituted with 2%*v*/*v* and 0.5%*v*/*v* ICP-MS grade nitric acid and hydrochloric acid in ultrapure water (18.2 mΩ·cm), respectively. Mg^2+^ concentration on both the apical and basolateral sides of the transwell was determined.

### 2.4. Gene Analysis

Real-time quantitative PCR was used to examine the mRNA expression levels of human TRPM6 and human TRPM7 from Caco-2 cells using human β-actin as a constitutively expressed gene for normalization. Total RNA was extracted from Caco-2 cell monolayers cultured on transwell inserts using a Norgen Biotek kit 35300 and a DNase I kit 25710 (Norgen Biotek, Thorold, ON, Canada). The qRT-PCR was performed using a Lightcycler 96 instrument (Roche, Switzerland) and a SensiFAST SYBR No-ROX one-step kit (Meridian Bioscience BIO72005, Cincinnati, OH, USA) according to the manufacturers’ protocols. Gene-specific primer pairs of human *TRPM6* forward primer *TGCCCTGGAACAAGCAATGTCAG* and reverse primer *CTTTTCATCAGCACAGCCCAAACC*, *TRPM7* forward primer *AGCATACAGAACAGAGCCCAACGG* and reverse primer *TTCCAACAGTGCCATCATCCACC,* and β-actin forward primer *CAGAGCAAGAGAGGCATCCT* and reverse primer *ACGTACATGGCTGGGGTG* were obtained from Eurofins, Luxembourg City, Luxembourg.

### 2.5. Statistical Analysis

The in vitro bioavailability data, including dextran and caffeine transport data, are mean ± standard errors of three independent experiments for each digested Mg^2+^ source, along with 3 analytical measurements for all measured values, *n* = 9. All data were analyzed in Sigma Plot 12.0 (Systat Software, Inc., Sigma Plot for Windows, San Jose, CA, USA). Data was analyzed using one-way analysis of variance (ANOVA) following the Grubbs test for outliers and normality test using the Shapiro–Wilk test. Tukey’s post hoc test was used for treatment group comparisons to assess for significant differences between variable mean values. Differences were significant at *p* < 0.05.

## 3. Results

### 3.1. Composition Analysis of Tested Magnesium Supplements

Compositional analysis of the supplements tested in this study is shown in Table 1 below. The analysis showed significant differences among supplements for all measured parameters. Magnesium oxide has the highest magnesium and inorganic content but the lowest organic content.

### 3.2. X-Ray Diffraction Pattern of Different Mg^2+^ Sources

**Figure 1:**
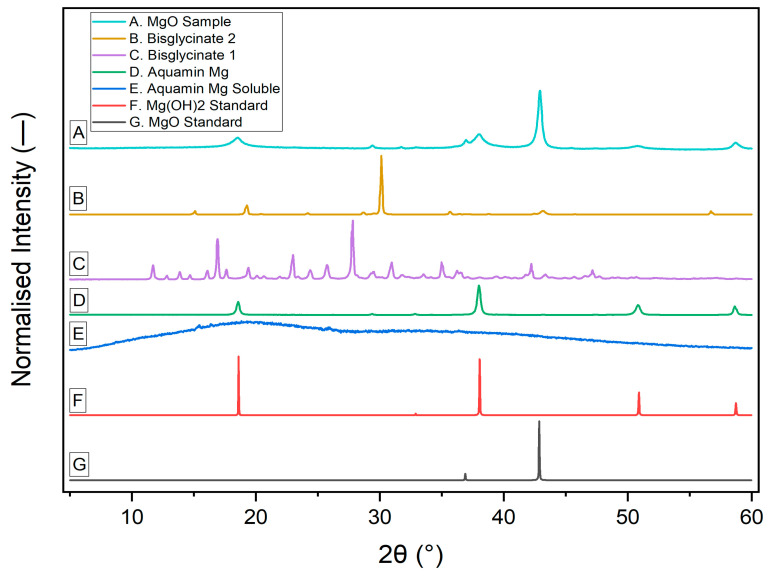
The XRD peaks corresponding to the MgO sample, bisglycinate 2, bisglycinate 1, Aquamin Mg, Aquamin Mg Soluble, Mg(OH)_2_ standard, and MgO standard are labeled as A, B, C, D, E, F, and G, respectively.

Figure 1 shows the X-ray diffraction (XRD) analysis of the tested magnesium supplements compared with reference standards. The figure shows the normalized intensity for seven samples: (A) MgO sample, (B) Mg bisglycinate 2, (C) Mg bisglycinate 1, (D) Aquamin Mg, (E) Aquamin Mg Soluble, prepared through enhanced solubilisation of Aquamin Mg, (F) Mg(OH)_2_ standard, and (G) MgO standard. MgO standard (G) exhibited sharp, well-defined peaks consistent with characteristics reflected by crystalline magnesium oxide. Similarly, Mg(OH)_2_ standard (F) displayed peaks at approximately 18°, 38°, and 50°, characteristic of pure Mg(OH)_2_. Both magnesium bisglycinates (B and C) displayed numerous sharp peaks characteristic of crystalline magnesium formulations. Differences in the number of peaks, intensity, and position suggest possible variations in the two formulations. Aquamin Mg, the precursor for Aquamin Mg Soluble, exhibited fewer and moderate intensity peaks, suggesting a mixture of crystalline and amorphous phases, while Aquamin Mg Soluble, a more soluble form of Aquamin Mg, showed a broad hump with only minor peaks, indicating predominantly amorphous phases. The MgO sample displayed broad peaks with some sharp features, indicating lower crystallinity compared to the MgO standard.

### 3.3. Surface Area

The BET surface area and total pore volume of the four magnesium supplements, along with Aquamin Mg (precursor for Aquamin Mg Soluble), are presented in Table 2. Across the samples, surface area ranged from 2 to 15 m^2^/g, while total pore volume varied between 0.002 and 0.207 cm^3^/g. All samples exhibited excellent isotherm fit quality (correlation coefficient ≈ 0.9999), with a slightly lower value for Aquamin Mg Soluble (0.9972), confirming robust adsorption model fits. Aquamin Mg Soluble displayed the lowest surface area (2 m^2^/g) and pore volume (0.002 cm^3^/g) compared to other samples. Although a large surface area per unit mass is generally advantageous for dissolution and bioavailability, the high bioavailability of Aquamin Mg Soluble observed in this study is likely attributable to its loss of crystallinity (as shown in Figure 1) during production, rather than changes in surface area. This structural transformation from a partially crystalline to a predominantly amorphous phase enhances solubility, compensating for its low surface area.

### 3.4. Evidence of Barrier Integrity and Active Transport Across Caco-2 Monolayer

**Figure 2:**
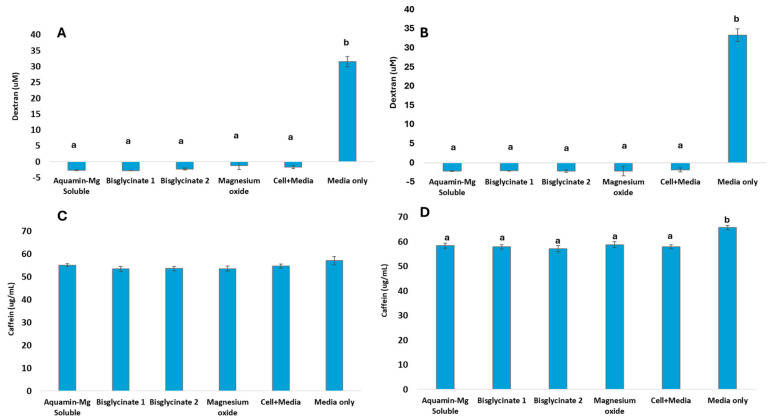
Caco-2 monolayer barrier integrity measured by the level of FITC Dextran (MW 3KDa) transported across the Caco-2 monolayer after 2 h of incubation in an experiment (**A**) involving bioavailability testing of Mg^2+^ sources digested with food, (**B**) bioavailability experiment of Mg^2+^ sources digested without food. (**C**) Testing of active transportation of caffeine across the Caco-2 cell monolayer after 2 h incubation with Mg^2+^ sources, digested with food material. (**D**) Mg^2+^ sources digested without food material. Data are means ± SE of three biological and three technical replicates, *n* = 9. Letters a and b are used to identify significant differences using ANOVA and Tukey post hoc test (*p* < 0.05).

The level of Fluorescein isothiocyanate-dextran (FTIC-Dextran, MW 3KDa) transported across the epithelial barrier created through the differentiation of Caco-2 cells is presented in Figure 2 below as a measure of barrier integrity. Figure 2A,B show that there was no transportation of dextran across Caco-2 cells, demonstrating an intact epithelial barrier in the transwells with Caco-2 cells compared to trans wells with media only. The level of caffeine transported across the Caco-2 cell monolayer as a measure of active transportation within the barrier is presented in Figure 2C,D. Figure 2C shows the amount of caffeine transported across the monolayer with Mg^2+^ compared to transportation across wells with media only in the experiment involving Mg^2+^ sources digested with food material prior to the Caco-2 experiment, while Figure 2D shows caffeine transportation in an experiment involving Mg^2+^ sources digested without food. The data demonstrates active transportation across the transwells containing Caco-2 monolayers.

### 3.5. Bioavailable Mg^2+^

**Figure 3:**
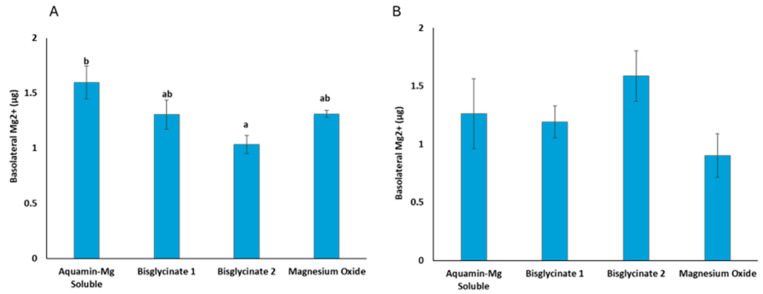
Mg^2+^ transported across the Caco-2 monolayer from (**A**) the Mg^2+^ sources digested with food material, and (**B**) the Mg^2+^ sources digested without food material. The results are means ± SE of three biological and three technical replicates, *n* = 9. ANOVA was performed to compare the means between the Mg^2+^ sources, followed by the Tukey test for multiple comparison. Digestion of the Mg^2+^ sources with food (**A**) significantly (*p* = 0.007) affects the amount of Mg^2+^ transported across Caco-2 monolayers. The Mg^2+^ sources digested without food material do not show significant differences (*p* = 0.253) in transportation across the Caco-2 monolayers. Letters a and b are used to identify significant differences using ANOVA and Tukey post hoc test (*p* < 0.05).

Two-way analysis of variance (Table 3) showed that food and Mg^2+^ source individually do not have a statistical influence on Mg^2+^ bioavailability. However, interaction between food and Mg^2+^ sources significantly affects Mg^2+^ bioavailability. The level of Mg^2+^ transported across the Caco-2 monolayer barrier into the basolateral side of the transwells is presented in Figure 3. The level of Mg^2+^ transported across Caco-2 cells varied among the tested Mg^2+^ sources digested with food material (Figure 3A), while there was no variation in Mg^2+^ transportation of the Mg^2+^ source digested without food material. The data demonstrates that a higher (*p* ≤ 0.004) level of Mg^2+^ from Aquamin Mg Soluble was transported across the monolayer barrier compared to magnesium bisglycinate when the different Mg^2+^ sources were digested with food material prior to Caco-2 monolayer application, while other Mg^2+^ sources showed comparable levels of Mg^2+^ transport.

### 3.6. TRPM6 and TRPM7 Expression

**Figure 4:**
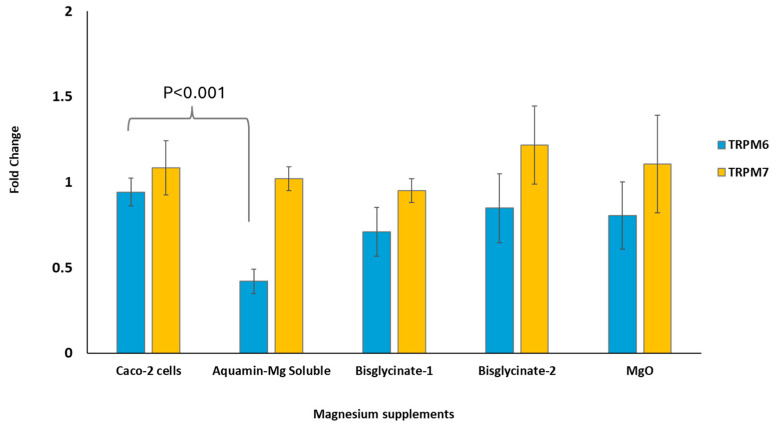
TRPM6 and TRPM7 expression in the Caco-2 cell monolayers treated with Mg^2+^ from the digestion protocol without food material measured by RT-qPCR. The results are expressed as means ± SE of three biological and three technical replicates, *n* = 9. ANOVA was performed to compare means within groups, followed by the Tukey test for multiple comparison. The supplements digested without food significantly (*p* = 0.037) impact TRPM6 expression but not TRPM7.

Figure 4 below shows the fold change in the expression of TRPM6 and TRPM7 in Caco-2 cells exposed to Mg^2+^ supplement digested without food material. The data suggested that treatment of Caco-2 cells with the Mg^2+^ supplement digested without food material has no effect on TRPM7 expression in the cells. Mg^2+^ from Aquamin Mg Soluble caused a significant (*p* < 0.001) downregulation of TRMP6 genes compared to the Caco-2 cells.

## 4. Discussion

The present study investigated the effects of Mg^2+^ source and the impact of food on the level of magnesium transported across differentiated Caco-2 cell monolayers, a well-established in vitro model of human intestinal epithelium [14]. Findings from this study demonstrate significant food × Mg^2+^ source interaction on Mg^2+^ transported across Caco-2 cell monolayers (Table 3). This suggests that Mg^2+^ bioaccessibility and, consequently, transportation across the intestinal barrier could be dependent on the surrounding particulate, biomolecule, and mineral composition of digesta. Although the main effect of food was not significant, the observed food × magnesium source interaction suggests that food components modulate magnesium bioaccessibility and transport in a Mg^2+^ source-specific manner. In the presence of food, Aquamin Mg (soluble) showed a significantly higher level of Mg^2+^ transport across the Caco-2 cell monolayer compared to bisglycinate 2, a commercial magnesium chelate, and a numerically higher level compared to bisglycinate 1 and MgO. There is no previous data comparing Aquamin Mg Soluble to magnesium glycinates. However, Felice et al. (2018) [8] reported that significantly higher level of Mg^2+^ was transported from Aquamin Mg compared to MgO. Felice et al. (2018) [8] had attributed this high bioavailability to improved bioaccessibility of Mg^2+^ from Aquamin Mg compared to MgCl and MgO, which was also reported by Dowley et al. (2024) [27].

The superior activity of Aquamin Mg Soluble reported in this study could be attributed to the presence of trace minerals in Aquamin Mg Soluble and its interaction with the food material. Food components such as phytate, oxalate, fiber, and proteins can reduce magnesium bioavailability by forming poorly soluble or insoluble complexes [30,31]. However, Mg^2+^ delivered as part of a multimineral complex (marine-derived magnesium), such as Aquamin Mg Soluble, does not behave as an isolated Mg^2+^ salt. Instead, the presence of additional trace minerals could cause competition for binding sites, thereby reducing the likelihood of selective Mg^2+^ sequestration, preserving competent transportable Mg^2+^ for epithelial uptake. Additionally, solubility of mineral supplements and consequent intestinal transport are strongly governed by physical properties such as crystallinity [32,33] and surface area [34,35]. While high surface area promotes rapid dissolution, reduced crystallinity lowers lattice energy, which facilitates quicker ion release under intestinal digestive conditions [32]. Though Aquamin Mg Soluble shows a much smaller BET surface area than the other samples, the material has increased solubility, likely due to the loss of crystallinity [27]. The reduced surface area of Aquamin Mg Soluble compared to Aquamin Mg could be a direct effect of the production process from Aquamin Mg, which results in loss of crystallinity (Figure 1).

The use of the Caco-2 monolayer in the transwell model in bioavailability studies has been broadly studied among researchers and is a non-invasive, rapid, and cost-effective method for screening mineral sources [9,14,15]. There are certain limitations associated with the models. The Caco-2 cells are derived from a tumor and may not fully represent the physiological conditions of intestinal cells. The model also lacks the presence of several endothelium components, such as a mucus layer, immune cells, and stroma cells, and may pose variability in barrier properties under different passage numbers or culture conditions [36,37]. However, studies have shown that a well-polarized Caco-2 cell can express some structural and functional characteristics of intestinal epithelial cells, such as microvilli, tight junctions, some enzymes, and transporters [14,36]. Also, efforts are being made to improve the Caco-2 model by means of automation and co-culture [14]. Improving this model will also involve incorporating an in vitro digestion protocol into the model, especially for bioavailability studies. This is in view of the fact that some in vitro bioavailability studies [8,15] with a digestion step have reported results that are comparable to clinical studies, while studies that failed to incorporate this protocol have reported contradictory results [9].

Intestinal absorption of Mg^2+^ takes place through paracellular and transcellular transport systems and is regulated in part by a group of proteins known as transient receptor potential melastatin (TRPM). Two members of the TRPM family, TRPM6 and TRPM7, are responsible for the regulation of divalent minerals, especially Mg^2+^ [21,22,24,38]. TRPM7 is ubiquitously expressed in most cells, whereas TRPM6 is mostly expressed in the kidney and intestinal cells [39]. In Caco-2 cells, TRPM6 is primarily localized to the apical membrane and contributes to Mg^2+^ entry into the cell, thereby facilitating transcellular Mg^2+^ absorption [24]. Previous studies have reported that TRPM6 gene expression can be influenced by intracellular Mg^2+^ status, with upregulation observed under conditions of Mg^2+^ deficiency and downregulation reported when intracellular Mg^2+^ is elevated [23,24]. In the present study, TRPM6 gene expression was significantly downregulated in Caco-2 cells treated with Aquamin Mg Soluble compared with untreated cells. This is in tandem with several in vivo studies that reported the sensitivity of TRPM6 gene expression to dietary Mg^2+^ status. Rondón et al. (2008) [24] demonstrated increased colonic TRPM6 expression in mice fed Mg^2+^-deficient diets, while van Angelen et al. (2013) [26] also reported elevated TRPM6 expression in mice consuming Mg^2+^-deficient diets. In contrast, Groenestege et al. (2006) [25] reported upregulation of TRPM6 in the colon of mice receiving Mg^2+^-enriched diets, although TRPM6 expression in the kidney was increased under Mg^2+^-deficient conditions. These discrepancies may reflect differences in experimental parameters, including animal age, dietary Mg^2+^ concentration, and duration of feeding regimens. For instance, Rondón et al. (2008) [24] fed mice diets ranging from 0.003 to 0.1% *w*/*w* Mg^2+^ for two weeks, while van Angelen et al. (2013) [26] used diets containing 0.0003% and 0.48% *w*/*w* Mg^2+^ over a similar duration, and Groenestege et al. (2006) [25] employed a 10-day feeding protocol with Mg^2+^ concentrations ranging from 0.005 to 0.48% *w*/*w*. Despite these variations, active transcellular Mg^2+^ transportation was suggested to be most relevant during low dietary Mg^2+^ intake [17], which is typically associated with increased TRPM6 expression as a compensatory mechanism.

While TRPM6 forms channels on the apical membrane of cells, TRPM7 is found mainly on the inner membrane of Caco-2 cells [40]. TRPM7 has been shown to be essential for maintaining cellular Mg^2+^ homeostasis, as TRPM7-deficient cells exhibit Mg^2+^ deficiency, impaired viability, and reduced proliferation, all of which can be rescued by Mg^2+^ supplementation [22]. Silencing TRPM7 using targeted siRNA has also been shown to significantly reduce Mg^2+^ influx in endothelial cells [41], supporting its role as a major Mg^2+^ permeable channel. In the current study, exposure of Caco-2 cells to different Mg^2^ supplements did not significantly alter the expression of the TRPM7 gene. This unchanged level of the TRPM7 gene in the Caco-2 cells treated with different magnesium forms is an indication of undisrupted Mg^2+^ homeostasis and cellular viability across treatments [42]. It is important to note that TRPM6 and TRPM7 activity are regulated at multiple levels, including post-transcriptional modifications, channel trafficking, and Mg^2+^ dependent feedback mechanisms [38,43,44].

The findings of this study are based on an in vitro Caco-2 monolayer model, which does not fully replicate the physiological complexity of the gastrointestinal tract. Therefore, the lack of an in vivo validation limits direct extrapolation to whole-body magnesium absorption and bioavailability. Also, the analysis was restricted to TRPM6 and TRPM7 gene expression, without assessment of protein abundance, cellular localization, or functional activity. Thus, given the extensive post-transcriptional regulation of Mg^2+^ dependent feedback controlling these channels, mRNA changes may not fully reflect functional Mg^2+^ transport. Future studies will incorporate an in vivo comparison, protein-level analysis, and functional assay such as transepithelial Mg^2+^ influx.

## 5. Conclusions

This study has demonstrated good transportation of Mg^2+^ from Aquamin Mg Soluble compared with commercial magnesium bisglycinates and oxide. Also, exposure of the different magnesium supplements digested with food to the Caco-2 cell monolayers was associated with altered expression of TRPM6 mRNA, with no changes in TRPM7 expression. These transcriptional responses may reflect cellular adaptation to Mg^2+^ availability rather than functional changes in transport activity. The use of a Caco-2 model, mRNA level analysis, and the absence of in vivo validation limit physiological interpretation. Therefore, future studies will incorporate functional transport assays and in vivo models to confirm relevance to intestinal Mg^2+^ absorption.

## Figures and Tables

**Table 1:** Compositional analysis of Mg^2+^ sources.

Parameter (%*w*/*w*)	Aquamin Mg	Bisglycinate 1	Bisglycinate 2	Magnesium Oxide
Inorganic	23.25 ± 0.05 a	26.92 ± 0.46 b	36.93 ± 0.13 c	79.27 ± 0.33 d
Organic	76.75 ± 0.06 d	73.08 ± 0.46 c	63.07 ± 0.13 b	20.73 ± 0.33 a
Mg^2+^	10.90 ± 0.50 a	12.11 ± 0.37 a	20.62 ± 1.96 b	40.82 ± 1.51 c
Other inorganic	12.30 ± 0.50 a	14.81 ± 0.37 b	16.31 ± 1.96 b	38.45 ± 1.51 c

Data are means ± SD of triplicate analysis. Letters a–d are used to identify significant differences among Mg^2+^ sources using ANOVA and Tukey post hoc test (*p* < 0.05).

**Table 2:** Surface area and pore volume of Aquamin Mg Soluble, Mg bisglycinate 1, Mg bisglycinate 2, MgO, and Aquamin Mg.

Sample Total Pore	Surface Area (m^2^/g)	Correlation Coeff	Pore Volume (cm^3^/g)
Aquamin Mg Soluble	2	0.9972	0.002
Mg Bisglycinate 1	9	0.9999	0.036
Mg Bisglycinate 2	7	0.9999	0.042
MgO	14	0.9999	0.207
Aquamin Mg	15	0.9999	0.079

**Table 3:** Two-way analysis of variance of the factors that affect magnesium bioavailability.

Source of Variation	Basolateral Mg^2+^ Transport (µg)
Food	*p* = 0.512
Source	*p* = 0.259
Food × Source	*p* = 0.026
Food	
Yes	1.31 ± 0.07
No	1.24 ± 0.08
Magnesium Source	
Aquamin Mg Soluble	1.43 ± 0.22
Mg Bisglycinate 1	1.25 ± 0.14
Mg Bisglycinate 2	1.31 ± 0.15
Magnesium Oxide	1.11 ± 0.11

*p*-values < 0.05 were considered statistically significant. *n* = 3 × 3.

## Data Availability

The original contributions presented in the study are included in the article.
